# Mechanisms of Cancer Resistance to Immunotherapy

**DOI:** 10.3389/fonc.2020.01290

**Published:** 2020-08-06

**Authors:** Rilan Bai, Naifei Chen, Lingyu Li, Nawen Du, Ling Bai, Zheng Lv, Huimin Tian, Jiuwei Cui

**Affiliations:** Cancer Center, The First Hospital of Jilin University, Changchun, China

**Keywords:** neoplasm, immunotherapy, resistance, mechanism, IFN-γ

## Abstract

Over the last decade, based on the extensive development of preclinical animal studies and clinical trials, the efficacy, and mechanisms of immunotherapy have been fully explored. Significant and lasting clinical responses with immunotherapy provide a new breakthrough treatment for a variety of refractory cancer histologies, which gradually change the treatment pattern of tumors. However, although immune checkpoint inhibitor drugs are promising for achieving longer-term efficacy, their benefits in the overall population are still very low, such as low frequency of response in some common tumor types such as breast and prostate, and heterogeneity in the degree of response among different tumor lesions in the same patient, making immunotherapy with many limitations and challenges. Most patients do not respond to immunotherapy or inevitably develop resistance to treatment after a period of treatment, manifesting with primary resistance or acquired resistance who initially respond to treatment. The mechanisms of tumor immune resistance are very complex and involve multiple aspects such as genes, metabolism, inflammation, and abnormal neovascularization. Currently, many mechanisms of immunotherapy resistance have been characterized, and more continue to be uncovered. These efforts can improve the quality of medical care for cancer diagnosis and treatment, which improve the quality of life of patients, and finally lead to accurate individualized treatment. This review discusses mechanisms of cancer immunotherapy resistance including tumor-intrinsic factors and tumor-extrinsic factors.

## Introduction

Over the last decade, based on the extensive development of preclinical animal studies and clinical trials, the efficacy, and mechanisms of immunotherapy have been fully explored. Significant and lasting clinical responses with immunotherapy provide a new breakthrough treatment for a variety of refractory carcinoma, which gradually change the treatment pattern of tumors. For example, the application of chimeric antigen receptor T-cell immunotherapy has become a new breakthrough therapy, as its success rate against refractory B lymphocytic leukemia is more than 90% (1). Another class of prominent therapies is immune checkpoint inhibitors (ICIs) therapy, which currently have been the first-line treatments for patients with non–small cell lung cancer (NSCLC), melanoma, and renal cell carcinoma; and they are also an option for some advanced and refractory tumor types, such as bladder tumors and head and neck tumors (2). However, although ICIs are promising for achieving longer-term efficacy, their benefits in the overall population are still very low, such as low frequency of response in some common tumor types, such as breast and prostate, and heterogeneity in the degree of response among different tumor lesions in the same patient (3), making immunotherapy with many limitations and challenges. Most patients do not respond to immunotherapy or inevitably develop resistance to treatment after a period of treatment, manifesting with primary resistance or acquired resistance who initially respond to treatment. At present, the biggest problem facing cancer immunotherapy is to deeply dissect its complex drug resistance mechanisms and adopt effective combination therapy strategies to overcome it. The mechanisms of tumor immune resistance are very complex and involve participation of genes, metabolism, inflammation, abnormal neovascularization, and other aspects. Currently, a variety of mechanisms of immunotherapy resistance have been explored in order to improve the efficacy of cancer immunotherapy and to expand the scope of its clinical applications, and more continue to be uncovered. This review discusses the mechanisms of cancer immunotherapy resistance including tumor-intrinsic factors, such as changes in antitumor immune response pathways and signaling pathways in tumor cells, as well as many changes of tumor cells participating in the formation of an inhibitory immunosuppressive microenvironment; and tumor-extrinsic factors, such as the local tumor microenvironment (TME) and host-related factors.

## Classification of Immunotherapy Resistance

Immunotherapy resistance is classified as primary resistance or acquired resistance. Primary drug resistance, also known as intrinsic resistance, represents a clinical situation in which a malignant tumor does not respond to immunotherapy (3). The incidence of primary resistance was as high as 60% in some cancer types when the clinical response rate was low (3). There is a form of primary resistance called hyperprogression (HPD) (4–6). Existing studies suggest that amplification of murine double minute (MDM) 2/4 gene (5, 7), epidermal growth factor receptor (EGFR) gene mutation (4), and the chromosome 11 region 13 (CCND1/FGF3/FGF4/FGF19) (4) are associated with the development of HPD. For example, overexpression of MDM2, as ubiquitin ligase, can perturb its regulation of wild-type (WT) p53, impede transcriptional activation domain of p53 gene, and lead to p53 inactivation by downregulation of ubiquitin-dependent p53 protein by proteases (4). Anti–programmed cell death-1/programmed cell death-ligand 1 (PD-1/PD-L1) inhibitors can upregulate interferon γ (IFN-γ) and activate the JAK-STAT signaling pathway, which lead to IFN regulatory factor 8 (IRF8) expression. Anchorage of IRF8 to the MDM2 promoter mediates its expression and thus may lead to HPD (4). Recently, several hypotheses for the development of HPD during immunotherapy were proposed by Champiat et al. (5) For example, blockade of immune checkpoints (i) has the potential to functionally stimulate regulatory T cells (Tregs), locally creating an immunosuppressive microenvironment, that is, enhanced compensation of negative regulatory signals further aggravates T-cell depletion; (ii) has the potential to lead to polarization of immunosuppressive cells, such as M2 macrophages, dendritic cells (DCs), or myelocytes, producing immunosuppressive cytokines; and (iii) has the potential to stimulate T_H_1- and T_H_17-mediated inflammatory responses or lead to activation of certain driver gene pathways to activate oncogenic pathways, thus creating conditions for accelerated tumor growth and resistance to immunotherapy. However, the specific mechanism of HPD needs further investigation because the sample size of HPD patients in these studies was small, there was a lack of control groups, and the measurement method of tumor growth rate had not been validated. In the future, prospective, large-scale clinical studies are needed (4).

Acquired resistance represents a clinical situation in which a tumor can initially respond effectively to immunotherapy but relapse or progress after a period of treatment (3). With the increasing use of ICIs, there has been a gradual increase in patients with acquired resistance, for example, in patients with advanced melanoma, approximately one-fourth to one-third relapse after treatment (8, 9). In addition, because of the principle that immunotherapy activates the autoimmune system, adaptive immune resistance is another newly proposed mechanism that distinguishes from traditional chemoradiotherapy and targeted therapy, representing a mechanism that a tumor can be recognized by immune system, but it can evade immunity by altering itself to adapt to immune attack (3). Here, under the dynamic regulation of the immune microenvironment and interaction between immune cell and cancer cell, adaptive resistance could occur as primary resistance, mixed responses, or acquired resistance (3).

## Mechanisms of Cancer Resistance to Immunotherapy

**Figure 1:**
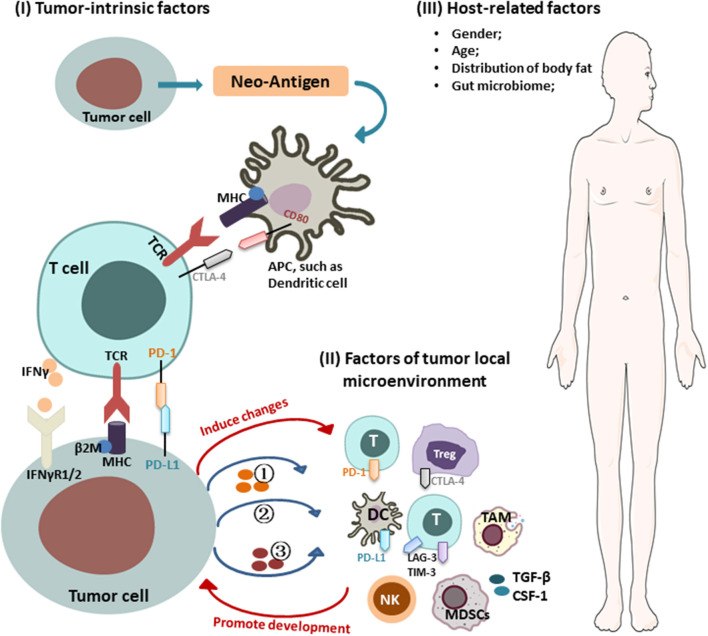
Mechanisms of cancer resistance to immunotherapy. Key mechanisms of cancer resistance to immunotherapy are briefly described in the figure, including: (I) Tumor-intrinsic factors: (i) alteration of antitumor immune response pathways: (1) aberrant expression of tumor antigens and changes in immunogenicity (e.g., TAA, TSA, antigenic drift, ER stress and autophagy, genes involved in mitochondrial metabolism); (2) alterations in the antigen presentation pathways [e.g., proteasome, transporters, MHC (loss of HLA expression, β2-M mutations leading to loss of HLA)]; (ii) alteration of signaling pathways (e.g., interferon γ and its associated-signaling pathway); (iii) forming immunosuppressive microenvironment: (1) secreting inhibitory molecules (e.g., exosome PD-L1, PD-L1 variant fragment); (2) functional gene mutations [e.g., Wnt/β-catenin signaling, PTEN/PI3K (phosphatase and tensin homolog/phosphatidylinositol 3-kinase) signaling, CDK4-CDK6 signaling, MAPK signaling, EGFR, KRAS, STK11, PAK4]; (3) altering metabolism in TME [e.g., hypoxic, expression of IDO, LXR, CD38/adenosine, CD73]; (II) factors of tumor local microenvironment: (i) immunosuppressive cells/molecules: immunosuppressive cells [e.g., MDSCs, Tregs, TAM]; activation of coinhibitory receptors (e.g., PD-L1, CTLA-4, TIM-3, TIGHT); inhibition of costimulatory receptor (e.g., CD28); (ii) abnormal neovascularization; (III) host-related factors: (i) gender; (ii) age; (iii) distribution of body fat; (iv) gut microbiome.

Intrinsic mechanisms of tumor immune resistance include changes in antitumor immune response pathways, alteration of signaling pathways in tumor cells, and other changes in tumor cells that lead to an inhibitory immunosuppressive microenvironment. Extrinsic factors involve local TME and host-related factors, which can synergize with tumor cells to promote their growth and resistance to ICIs (Figure 1).

### Tumor-Intrinsic Factors of Resistance to Immunotherapy

#### Alterations of Antitumor Immune Response Pathways Contribute to Resistance to Immunotherapy

The process of an immune response includes the processing of tumor-associated antigenic peptides by antigen-presenting cells (APCs), presentation to CD8^+^ T cells, stimulation of T-cell proliferation and activation, and activation of T cells in TME that kill tumor cells (10). Here, APCs include professional cells such as DCs, monocyte–macrophages, B lymphocytes, and other non-professional APCs such as endothelial cells, fibroblasts, epithelial, mesothelial cells, and so on (11). Any change within antitumor immune response pathways has the potential to lead to resistance to immunotherapy.

##### Aberrant expression of tumor antigens and changes in immunogenicity

High tumor mutation burden (TMB) (non-synonymous mutations), microsatellite instability, and mismatch repair deficiency (dMMR) are tumor-intrinsic features associated with antitumor immune responses and responses to ICIs (12, 13). These responses are closely related to increased neoantigen [tumor-associated antigen (TAA) and tumor-specific antigen (TSA)] formation, which confers higher immunogenicity and increased T-cell infiltration, as demonstrated in a variety of tumors (14–17). However, tumor cells often inhibit T-cell activation by reducing or losing antigen expression (18) and regulate autoantigenicity by means of endocytic antigens or antigen shedding to mediate immune escape. For example, analysis of allele frequency plots in resistant patients showed loss of three neoantigens (C17orf78, HSD17B1,WNK4) on chromosome 17 (19, 20). In addition, the host can selectively eliminate TSA-expressing cells and to some extent promote the generation of tumor antigen-losing variants (21). Tumor cells also can undergo “antigenic drift” like viruses, leading to epitope mutations that alter the antigenicity of tumors, which subsequently escape T cell–mediated attack (22). Endoplasmic reticulum (ER) stress and autophagy determine the immunogenicity of cell death in tumors (23, 24). The abundance of LC3B puncta, representing active autoghagy machinery in the cytosol of tumor cells, is related to infiltration of tumor-infiltrating lymphocytes (TILs) and a good clinical outcome, whereas tumor cells do not respond to ER stress, or autophagy induction may result in the resistance to ICIs (25, 26). In addition, antigen presentation was inhibited by low expression of genes involved in mitochondrial metabolism. Analysis of sensitive patients treated with TIL and anti-PD1 showed a high concentration of proteins involved in mitochondrial metabolism-related pathways and of proteins involved in antigen presentation, whereas the process of antigen presentation was significantly reduced when mitochondrial energy metabolism was inhibited by inhibitors (27).

##### Alterations in the antigen presentation pathways

Disruption of the antigen presentation signal pathway can render T cells unable to activate and lead to tumor immune escape, including mutations that interfere with the proteasome, transporters involved in antigen processing, and changes in the structural composition of major histocompatibility complex (MHC) itself. This has been demonstrated in resistance mechanisms in patients with metastatic melanoma (28) and advanced prostate cancer (29). The abnormalities of MHC molecules are broadly divided into structural defects due to gene mutations, such as mutations within the receptor-binding domain of MHC cells (30), and defects in regulatory mechanisms due to epigenetic changes (31). These epigenetic changes are often associated with the downregulation of transporters associated with antigen processing, the downregulation of low-molecular-mass proteins and methomyl proteins, and the inactivation of class I MHC gene transcription (31). For example, the loss-of-function mutation of β2-microglobulin impairs the folding, transport, and stable expression of MHC-I on the cell surface (8, 32, 33). In addition, in certain malignancies, such as advanced multiple myeloma, tumor cells can escape the lysis mediated by cytotoxic T lymphocyte (CTL) and natural killer (NK) cell by overexpressing non-classical MHC-I molecules (e.g., HLA-G), leading to the development of immune escape (34).

#### Alterations of Signaling Pathways in Tumor Cells Contribute to Resistance to Immunotherapy

Interferon γ is a cytokine produced and secreted by effector T cells (T_effs_) and APC. It leads to JAK2 activation by binding to IFN-γ receptors 1/2 (IFNGR1/2) (2). IFNGR1/2 can subsequently interact with signal transducers and activators of transcription-1 (STAT1) to regulate the downstream cascade (8) and activate the transcriptional activity of IRF1, and ultimately cause the expression of antigen-presenting molecules by pathways such as upregulation of MHC molecules. Interferon γ plays a role in immune escape by increasing the expression of PD-L1 on the surface of tumor cells (35); it can also induce the production of chemokines CXCL9 and CXCL10 and promote the recruitment of CXCR3+ lymphocytes and other immune cells around tumor cells to exert antitumor immune effects (36); in addition, IFN-γ can directly exert antitumor cell proliferation and proapoptotic effects by binding to receptors on the cell surface and triggering a series of events that inhibit tumor cell growth and promote tumor cell death (35). In patients receiving immunotherapy, tumor cells can downregulate or alter IFN-γ signaling pathways such as loss-of-function alleles of genes encoding for JAK1/2 and changes in STAT1 to escape the influence of IFN-γ (37), resulting in resistance. Zaretsky et al. (8) demonstrated that patients with melanoma who developed resistance to PD-1 therapy acquired loss-of-function mutations in JAK1/2. Although tumor cells are still recognized by T cells, their JAK1/2 mutations render them insensitive to the antiproliferative effects of IFN-γ and lack of IFN-γ-induced surface expression of both PD-L1 and MHC class I. Similarly, tumor analysis of patients resistant to treatment with the anti–cytotoxic T-lymphocyte–associated protein 4 (CTLA-4) agent, ipilimumab, revealed that mutations in the IFN-γ pathway genes, IFNGR1/2, JAK1/2, and IRF1, could suppress tumor cells in response to IFN-γ signaling (38). This favors tumor escape from T-cell immunity, thereby conferring resistance to anti–CTLA-4 therapy.

**Figure 2:**
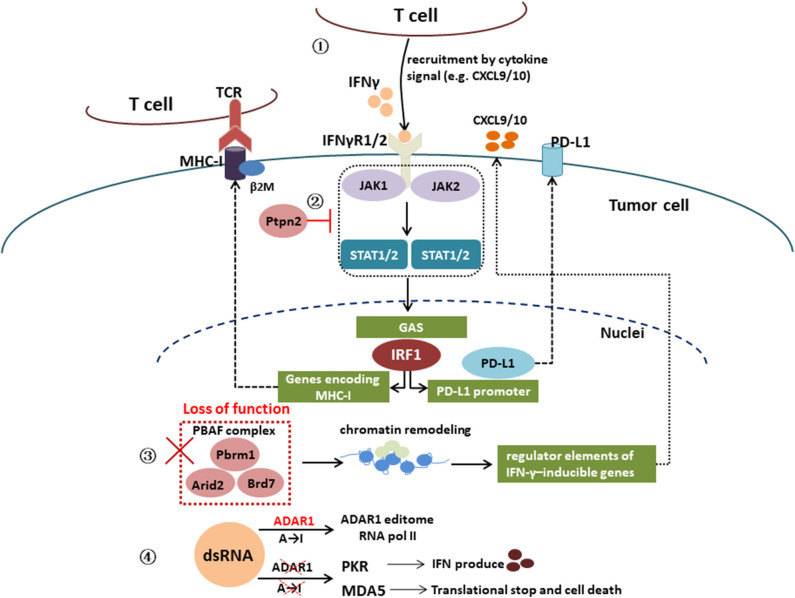
Interferon γ signaling pathway. IFN-γ signaling pathway: IFN-γ → IFNγR1/2 → JAK1/2 → STAT1/2 → IRF1 → genes encoding MHC-I or PD-L1 promoter; activating mutations in tyrosine–protein phosphatase non-receptor type 2 (Ptpn2) negatively regulates JAK1 and STAT1 signaling; loss of function of PBAF complex unique genes (Pbrm1, Arid2, and Brd7) increased chromatin reachability to transcription regulator elements of IFN-γ-inducible genes within tumor cells and subsequently increased production of CXCL9/CXCL10 chemokines, leading to more efficient recruitment of T_effs_ to tumor tissue; loss of function of the RNA-editing enzyme ADAR1 in tumor cells could reduce A-to-I editing of IFN-inducible RNA species and elicit a sensing response of melanoma differentiation-associated protein 5 (MDA5) and PKR to dsRNA ligand, which leads to tumor inflammation and growth inhibition, respectively.

Furthermore, genetic alterations associated with the IFN-γ signaling pathway can also affect immune resistance. In a CRISPR screen, activating mutations in tyrosine–protein phosphatase non-receptor type 2 (Ptpn2), which negatively regulates JAK1 and STAT1 signaling, has also been associated with primary resistance to PD-1 blockade via resistance to IFN-γ (39). Deletion of Ptpn2 via CRISPR-Cas9 genome editing was able to restore IFN-γ sensitivity in a melanoma model (39). Recent studies suggest that inactivating mutations in a mammalian analog of the chromatin remodeling SWI/SNF complex and unique genes of the PBAF complex (Pbrm1, Arid2, and Brd7) leads to sensitivities to ICIs (40, 41). Loss of function of the PBAF complex increased chromatin reachability to transcription regulator elements of IFN-γ-inducible genes within tumor cells and subsequently increased production of CXCL9/CXCL10 chemokines, allowing more efficient recruitment of T_effs_ to tumor tissue (42). In human cancers, expression of Arid2, and Pbrm1 is related to expression of T-cell cytotoxicity genes, which confirmed in Pbrm1-deficient murine melanomas with strongly infiltrated by cytotoxic T cells and responsive to immunotherapy (41, 42). In addition, double-stranded RNA (dsRNA) editing enzyme, adenosine deaminase acting on RNA (ADAR1) protein can block the IFN-γ signaling pathway and lead to immunotherapy resistance. Loss of function of ADAR1 in tumor cells could reduce A-to-I editing of IFN-inducible RNA species and elicit a sensing response of melanoma differentiation-associated protein 5 (MDA5) and PKR to dsRNA ligand. This leads to tumor inflammation and growth inhibition, respectively, and profoundly sensitizes tumors to immunotherapy, overcoming resistance to ICIs (43). Figure 2 is a schematic of IFN-γ signaling pathway.

#### Tumor Cells Participate in the Formation of an Immunosuppressive Microenvironment

##### Tumor cells contribute to resistance to immunotherapy by secreting inhibitory molecules

Tumor cells also secrete exosomes carrying inhibitory ligand, PD-L1, to peripheral blood to exert inhibitory effects on T cells outside of TME, which include inducing apoptosis of CD8^+^ T cells, inhibiting proliferation of CD4^+^ T cells, upregulating the suppressive function of Tregs, and downregulating the expression level of NKG2D on the surface of NK cells (44). The above pathways finally promote immune escape of tumor cells (44). A study showed that huntingtin interacting protein 1 related protein (HIP1R) targets PD-L1 to lysosomal degradation to change cytotoxicity mediated by T cells, but inactivation of HIP1R in tumor cells caused the expression of PD-L1 and inhibitory cytotoxicity mediated by T cells (45). A designed peptide (PD-LYSO) that combines PD-L1–binding sequence of HIP1R and lysosome-sorting signal can effectively deplete the PD-L1 expression on the surface of tumor cells (45). In addition, RNA sequencing analysis of PD-L1 inhibitor-resistant NSCLC patients revealed the presence of a PD-L1 variant fragment (v242 and v229, which retain the PD-1–binding domain) *in vivo* and confirmed its inhibitory effect on T-cell activity (46).

##### Functional gene mutations in tumor cells contribute to resistance to immunotherapy

Aberrant Wnt/β-catenin signaling is observed in a variety of tumor tissues that correlates with tumor cell genesis, invasiveness, and metastatic potential and is conducive to tumor protects itself from immune system–mediated oncolysis (47). In an experimental study, Spranger et al. (48) confirmed that the recruitment of CD103^+^ DC and infiltration of T cells into the TME can be prevented by tumor-intrinsic β-catenin activation though decreasing CCL4 expression. As DCs are prevented from migrating into the TME, no antigen can be presented to T cells, which thereby inhibits the activation and cytotoxic effects of T_effs_. Enhanced MAPK signaling could impair the infiltration and function of TILs through the expression of vascular endothelial growth factor (VEGF) and a variety of inhibitory cytokines such as interleukin 8 (IL-8) and many secreted proteins (49). Under these conditions, induction and maintenance of Tregs eventually lead to tumor immune escape. Several studies in mouse models have shown that MAPK inhibitors enhance the function of TILs, IFN-γ signaling, expression of MHC-1, and PD-L1 levels, thereby promoting tumor cell killing (50–52). Acquired resistance to BRAF inhibitors and MAPK inhibitors during targeted therapy of tumors can modulate the immune microenvironment, leading to depletion of TILs, which in turn leads to PD-1/PD-L1 inhibitor cross-resistance (53). Loss of the tumor suppressor phosphatase and tensin homolog (PTEN), which leads to activation of phosphatidylinositol 3-kinase (PI3K) signaling, has also been associated with release of anti-inflammatory cytokines, such as CCL2 and VEGF, reduced infiltration of CD8^+^ T cells in tumors, and decreased IFN-γ and granzyme B expression, conferring resistance of PD-1 blockade therapy to tumors (54). In addition, PTEN-deficient tumor cells tend to be less immunogenic. The study of glioblastoma tissue specimens has shown that T cells lyse PTEN WT tumor cells more efficiently than PTEN-mutant tumor cells and that reduced lysis is associated with increased receptor expression in B7-H1 cells (55). Oncogenic CDK4-CDK6 signaling also contributes to the immunosuppressive microenvironment by decreasing sensitivity to dsRNA by antigen presentation, activation of IFN target gene and DNA methyltransferase (DnMt1) (56).

Multiple clinical trials have shown that prolonged overall survival (OS) could be observed in the entire study population and in the subgroup with WT- EGFR, but no significant improvement in OS in the subgroup with EGFR mutation (EGFRm) (15, 57–60). Some basic experiments showed that EGFR mutation can upregulate PD-L1 expression to promote an immunosuppressive TME, such as the IL-6/JAK/STAT3 pathway (61). On the other hand, it is thought that patients with driver gene mutations such as EGFR, ALK, ROS1 fusion, BRAF fusion, and so on, already have one dominant gene, so the overall TMB of these patients is low and unlikely benefits from immunotherapy. However, patients with KRAS mutations benefitted more from ICIs, correlating with high TMB and elevated PD-L1 expression (62). Another study demonstrated that KRAS mutations could induce the upregulation of PD-L1 expression through p-ERK but not p-AKT signaling, which mediates immune escape of human lung adenocarcinoma (LUAD), and thus blockade of the PD-1/PD-L1 pathway could reverse T-cell apoptosis to promote antitumor effect in a coculture system (63). STK11 genomic mutation is another factor found to result in immune resistance in NSCLC (64), which is significantly more observed in tumors with moderate/high TMB and negative PD-L1 expression. In LUAD, patients with KRAS mutations and comutations in STK11 gene had inferior clinical outcomes and resistance to PD-1 blockade therapy (64). The expression of p21-activated kinase 4 (PAK4) is correlated with infiltration of T cells and DCs, which may mediate resistance to cancer immunotherapy (65). PAK4 gene deletion promotes the infiltration of CD8^+^T-cell and reverses resistance to anti–PD-1 in a mouse model, and further, compared with anti–PD-1 alone, combination strategy with PAK4 inhibitor significantly improved antitumor effects (65).

Tumor primary resistance exhibits transcriptional features, known as key features of IPRES or primary PD-1 antibody resistance, including related regulatory genes of mesenchymal transformation, angiogenesis, extracellular matrix remodeling, and so on. (66) This shows simultaneous upregulation of several genes, including epithelial–mesenchymal transition–related genes (AXL, WNT5A, ROR2, TWIST2, FAP, and TAGLN), VEGF pathway genes (IL10, VEGFA, and VEGFC), macrophage chemotaxis genes (CCL2, CCL7, CCL8, and CCL13), and so on (66).

##### Tumor cells contribute to resistance to immunotherapy by altering metabolism in TME

Tumor cells adapt to their own high-energy demands through a series of metabolic alterations, called tumor metabolic reprogramming (67). The produced lactate by the “Warburg” effect can stimulate the production of hyaluronic acid and the expression of CD44, which contributes to tumor metastasis (68). Meanwhile, a hypoxic, acidic TME caused by massive aerobic glycolysis inhibits normal metabolism of immune cells and T-cell function (69, 70). A study has shown that mitigation of TME hypoxia can improve the infiltration level of T cells. And when in combination with ICIs, it can lessen the drug resistance problem of “cold tumors” (71), which were defined as no or very little infiltration of CD3^+^ and CD8^+^ lymphocytes in the center and border area of the tumor, such as prostate cancer (72). In addition, glucose consumed by tumor cells can limit T-cell metabolism in the TME, which leads to inhibition of mTOR activity, intracellular IFN-γ production, and glycolytic capacity in T cells (73).

In recent years, more and more researchers believe that the catabolism of tryptophan in TME is a key factor responsible for the inhibition of antitumor immune responses. Tryptophan metabolism of T-cell is mediated by tumor cells through high expression of indoleamine 2,3-dioxygenase (IDO) and tryptophan-2,3-dioxygenase (TDO), which can convert tryptophan into kynurenine and its metabolites (74). Tryptophan is a crucial amino acid for the activation of T cell, and its depletion allows intracellular accumulation of large amounts of uncharged tryptophan transfer ribonucleic acid and activation of general control non-depressible 2 (GCN2) in cells, which terminates protein translation and activates T_effs_ (74). These processes can inhibit the clonal proliferation of T cells and induce T-cell inactive and apoptosis (74). Meanwhile, kynurenine can bind AhR transcription factors to stimulate the proliferation of Tregs and further inhibit the function of T_effs_ (75). Holmgaard et al. (76) demonstrated that when the IDO inhibitor 1-methyltryptophan (1MT) was combined with CTLA-4 treatment in mice with B16 melanoma that was resistant to CTLA-4 treatment, the tumors showed treatment sensitivity, suggesting that IDO inhibition could be a potential adjuvant therapeutic strategy for immunotherapies to reduce the development of resistance (76).

Cholesterol metabolism in tumor cells modulates the immune response. Cholesterol oxidation produces large amounts of epoxycholesterol and hydroxycholesterol, which can bind to the liver X receptor (LXR) as a ligand (77). Activated LXR signaling pathway has immunosuppressive effects that can attenuate the proliferative capacity of lymphocytes, stimulate the maturation and differentiation of inhibitory immune cells T_H_17, and inhibit maturation and migration of DCs (78) so that T cells cannot be activated by antigens. Recent evidence suggested that in murine melanoma growth and metastasis, inhibition of cholesterol esterification enhanced the inhibitory effect of CD8^+^ T cells, which is also observed in studies using avasimibe, an inhibitor of the key cholesterol esterase acetyl-CoA acetyltransferase (ACAT1) (79, 80).

Tumor cells produce adenosine by upregulating CD38. Activated adenosine receptor pathway can inhibit T-cell proliferation and cytotoxic function by binding to A2A receptors on T cells (81) and promote their metastasis via A2B receptors on tumor cells (82), resulting in acquired resistance. Immune checkpoint inhibitors combined with adenosine receptor antagonists can relieve the inhibitory effect of CD38 on tumor cells on CD8^+^ T-cell function. In addition, CD73, an enzyme that dephosphorylates adenosine monophosphate to form adenosine can inhibit immune function, promote tumor cell metastasis (83), and stimulate angiogenesis (84). High expression of CD73 is associated with poor prognosis in different tumor types (85). CD73 is also a potential biomarker for anti–PD-1 therapy and is associated with resistance to ICI therapies (86).

### Tumor-Extrinsic Factors of Resistance to Immunotherapy—Local Microenvironment

There are various immune and stromal cells and cytokines within TME, which can affect the response to immunotherapy. The changes of immunosuppressive cells, immunosuppressive cytokines, coinhibitory receptors, and costimulatory receptors in the TME can destroy the antitumor immune response, which are the important mechanisms mediating resistance to immunotherapy.

#### Immunosuppressive Cells and Molecules in TME Contribute to Resistance to Immunotherapy

T cells in the TME exhibit a state of exhaustion, and the epigenetic stability of exhausted T cells limits the remodeling of T cells and the persistence of responses to PD-1 inhibitor therapy. Regulatory T cells are immunosuppressive cells that control autoimmune responses. Regulatory T cells can inhibit the activation and proliferation of T_effs_ by various mechanisms, such as inhibiting MHC molecules and costimulatory molecules (CD80 and CD86) on the surface of APCs and inhibiting the maturation of APCs, as well as attenuating their interactions with T cells (87). Regulatory T cells also can directly kill T cells and APCs by secreting perforin and granzymes and inhibit the activation and proliferation of T cells by secreting inhibitory cytokines [e.g., transforming growth factor β (TGF-β), IL-10, IL-35] and depleting γc cytokines (88). Myeloid-derived suppressor cells (MDSCs), a type of cell of myeloid origin with potent immune-suppressive activity, consist of two different types of cells termed granulocytic or polymorphonuclear, with a morphology similar to neutrophils, and monocytic, with a phenotype and morphology similar to monocytes (89, 90). Thus, phenotypic characteristics alone are not sufficient to determine cells as MDSCs. Myeloid-derived suppressor cells were implicated in various aspects of immune regulation, not only tumor, but were also involved in diseases including infection, chronic inflammation, autoimmune diseases, and so on (91). In TME, MDSCs can contribute to tumor progression by promoting its immune escape, cell growth, and angiogenesis, as well as invasion and metastasis (92), which as a class of regulatory cells can inhibit T-cell function by producing immunosuppressive metabolites (reactive nitroxide intermediates), immunosuppressive cytokines (e.g., TGF-β, IL-10), immunoactive enzymes (ARG, IDO, aminopeptidase), and immunosuppressive PGE2 (93). For example, MDSCs are major contributors to local immunosuppression in head and neck cancers and can contribute to patient resistance to ICIs. Limiting MDSCs function is a plausible strategy to enhance the response to CTLA-4 inhibitors in these patients (94).

Tumor-associated macrophages (TAMs) massively infiltrate around tumor cells due to action of colony-stimulating factor 1, VEGF, and certain chemokines (95). In the development of early tumors, TAM induces chronic inflammatory carcinogenesis by releasing proinflammatory mediators, such as reactive nitrogen/oxygen intermediates, IL-6, and IL-1β (96). Tumor-associated macrophage also increases tumor angiogenesis and promotes tumor invasion and metastasis by producing proangiogenic factors such as VEGF, EGF, and MMP (97). In addition, TAM can inhibit the antitumor response of T cells by overexpressing PD-L1, ARGI, PGE2, and TGF-β and promote the cellular accumulation of Tregs by expressing the chemokine CCL22 (98). A study showed that Fc domain glycan of the drug and Fcγ receptor (FcγR) expressed by the host bone marrow cells could determine the ability of PD-1 TAMs to capture anti–PD-1 drugs from the surface of T cells, which leads to PD-1 inhibitor resistance (99). In mouse experiments, blocking FcγR prior to anti–PD-1 drug administration can significantly prolong the binding of the monoclonal antibody to CD8^+^ tumor-infiltrating T cells and improved the antitumor effect of immunotherapy (99). Besides PD-1, immune cells can express other inhibitory receptors such as CTLA-4, T-cell immunoglobulin 3 (TIM-3), ITIM domain protein (T-cell immunoglobulin and ITIM domain, TIGHT) (100) to mediate tumor immune resistance. Coexpression of multiple immune checkpoints is associated with severe exhaustion of T-cell status. Thommen et al. (101) showed that increased coexpression of multiple immune checkpoints, such as PD-1, TIM-3, CTLA-4, and LAG-3, can promote the progressive failure of T cells and further mediate the development of resistance to ICIs. Therefore, monoclonal antibodies targeting these novel immune checkpoints can be used as potentially effective antitumor therapeutics.

Recently, CD28 has been found to play a role in the mechanism of immunotherapeutic resistance, and its therapeutic efficacy as a new target for immunotherapeutic strategies has been extensively studied. In contrast to CTLA-4, CD28 is a costimulatory receptor that interacts with CD80/86 to modulate immune function and can activate multiple mechanisms that promote immune responses (2). Activation of CD28 via Lck tyrosine kinase, PI3K pathway, protein kinase C, and so on, can activate a variety of transcription factors such as activator protein 1 and nuclear factor κB (NF-κB), which are essential for the activation and survival of IL-2 and T cells, which are essential for IL-2 and the activation and survival of T cells (2). Furthermore, recent evidence shows that CD28 may be related to intact T-cell receptor expression and actin cytoskeleton remodeling via Vav-1, cofilin-1, and RLTPR, thereby enhancing T-cell activation (2). Kamphorst et al. (102), using mice treated with B7 antibody and PD-L1 inhibitor, demonstrated that inhibition of CD28 leads to the progression of colon cancer and inhibition of T-cell expansion in CD28 knockout mice using PD-L1 inhibitor alone. In the future, situations that enhance of CD28 receptor expression may lead to reactivation of exhausted T cells and enhancement antitumor immune responses by enhancing CD28 receptor expression.

#### Abnormal Neovascularization in TME Contributes to Resistance to Immunotherapy

The massive production of proangiogenic factors such as VEGF in tumor tissues, as immunosuppressive factors, can inhibit DCs maturation by directly activating the NF-κB pathway, and on the other hand, they also can lead to abnormal and dysfunctional tumor vessel morphology (103). Vascular maldistribution and abnormal distribution of adjacent endothelial cells and pericyte structures can lead to impaired vascular perfusion and increased vascular permeability. Meanwhile, tumor-associated endothelial cells can express low levels of cell adhesion molecules, resulting in blocked entry of immune cells into tumor tissue (104) and further resulting in tissue hypoxia, acidosis, and necrosis. This aggravates the hypoxic acidic state of TME, which in turn inhibits a series of functions of immune cells, and promotes the accumulation of immunosuppressive cells such as MDSCs, TAM, and Tregs and the secretion of immunosuppressive factors such as VEGF, TGF-β, and IL-10 to promote angiogenesis and eventually aggravate vascular abnormalities (105, 106). In addition, a hypoxic acidic environment can also induce the upregulation of PD-L1 on tumor cells and CTLA-4, TIM-3, and PD-L1 on the surface of immunosuppressive cells, which help tumor cells escape the killing mediated by T cells and NK cells and ultimately promote tumor development (107).

### Tumor-Extrinsic Factors of Resistance to Immunotherapy—Host-Related Factors

Intrinsic factors of cancer patients, including age, gender, hormone, diet, intestinal flora, and other factors may affect the efficacy of immunotherapy and drug resistance. Aging is associated with restricted immune function with significant effects on both innate and adaptive immune responses (108). However, results of more large-scale randomized controlled trials (RCTs) and retrospective cohort analysis indicated that the application of ICIs had similar efficacy and safety between elderly patients and young patients (109). A meta-analysis including 20 RCTs of ICIs (*n* = 11,351) reported OS according to gender in patients with advanced tumors, 67% in men and 33% in women. The study showed that pooled OS hazard ratio of male patients was 0.72 [95% confidence interval (CI), 0.65–0.79] compared with the control group, whereas that of female patients was 0.86 (95% CI, 0.79–0.93), and gender difference in the efficacy ICIs was significant (*P* = 0.0019) (110). Obese mice also showed a significantly better response to anti–PD-1 without significant toxic side effects, and this effect was also reproduced in multiple cancer populations receiving ICIs, with higher body mass index (>30 kg/m^2^) patients showing longer PFS and OS; moreover, benefit was more pronounced in males. Differences in body fat distribution and hormones between males and females may be important factors related to the effect of immunotherapy (111).

In addition, gut microbiome is an extensively studied host factor that can influence responsiveness and resistance to ICIs. It also might be associated with cross-reactivities between microorganisms and tumor antigens, which could increase the presentation of antigens and the production of inflammatory cytokines of DCs (112, 113). The responses of T cells after treatment with ICIs are associated with *Bacteroides thetaiotaomicron* and *Bacteroides fragilis* in the intestine. Recent evidence suggests that CTLA-4 blockade can induce the internal accumulation of distinct *Bacteroides* species such as *B. fragilis* in the mucus layer, which further elicits superactive and IL-12–dependent immune response effect of T_H_1 cells that favors the whole body against tumors (114). Injection of anti–CTLA-4 inhibitors into metastatic melanoma patients and analysis of their gut microbiome composition found three different microbiome clusters (“enterotypes”) segregated by *Bacteroides* and *Prevotella* species. Transplantation of fecal microbiota from these patients into germ-free tumor-bearing mice revealed that mice enriched with immunogenic *Bacteroides* species restored the efficacy of CTLA-4 inhibitor, whereas mice enriched with tolerogenic *Bacteroides* species are completely resistance to the monoclonal antibodies (114).

## Future Directions

In this review, we summarized mechanisms of resistance to immunotherapy, although the complex resistance mechanisms to immunotherapy are still not well-understood. The gradual elucidation and in-depth exploration of new immune resistance mechanisms contribute to the discovery of new therapeutic targets and continue to expand the scope of clinical applications of cancer immunotherapy. However, considering that only a minority of patients with ICIs can achieve a durable response, multimodal treatment strategies in addition to combination therapy should be developed to improve patient clinical outcomes and overcome the development of resistance. It is essential to explore effective predictive biomarkers, such PD-L1 expression, TIL status, and assessment of TMB and neoantigen, which have been explored so far. However, because of the complexity of the antitumor immune response and tumor heterogeneity among different patients, there are currently no suitable, widely and uniformly biomarkers to predict clinical benefits. Nevertheless, this exploration can help screen immunotherapy-dominant populations, develop personalized precise diagnosis and treatment programs, predict the efficacy of treatment, and adjust the treatment regimen in a timely manner. Exploring effective predictive biomarkers and developing effective combined strategies or other multimodal approaches are areas of intense study and remain considerable clinical challenges. These efforts can improve the quality of medical care for cancer diagnosis and treatment, which improve the quality of life of patients and finally lead to accurate individualized treatment. In the future, to clarify targetable, heterogeneous mechanisms of drug resistance, it is necessary to conduct sequential tumor biopsies and peripheral blood sampling of tumor patients during treatment and analyze comprehensively in combination with multiple factors or use new technologies, such as whole-genome sequencing, single-cell sequencing, and epigenetic analysis to identify characteristic drug resistance sites or subclones. With continuous in-depth exploration of mechanisms of resistance to immunotherapy, immunotherapy might be applied to treat a wider range of patients with tumor.

## Author Contributions

RB reviewed the literature, analyzed, and wrote the paper. NC, LL, ND, LB, ZL, and HT consulted the literature, reviewed, and modified the article. JC put forward valuable comments on the article, reviewed, and edited it. All authors read and approved the final manuscript.

## Conflict of Interest

The authors declare that the research was conducted in the absence of any commercial or financial relationships that could be construed as a potential conflict of interest.

## Abbreviations

ICIs: immune checkpoint inhibitors
PD-1/PD-L1: programmed cell death-1/programmed cell death-ligand 1
NSCLC: non–small cell lung cancer
TME: tumor microenvironment
HPD: hyperprogression
MDM2/4: murine double minute 2/4
EGFR: epidermal growth factor receptor
Tregs: regulatory T cells
IFN-γ: interferon γ
DCs: dendritic cells
TGR: tumor growth rate
APCs: antigen-presenting cells
TMB: tumor mutation burden
dMMR: mismatch repair deficiency
TAA: tumor-associated antigen
TSA: tumor-specific antigen
ER: Endoplasmic reticulum
TILs: tumor-infiltrating lymphocytes
MHC: major histocompatibility complex
T_effs_: effector T cells
STAT1: signal transducers and activators of transcription-1
IFNGR1/2: IFN-γ receptor 1/2
IRF1: interferon regulatory factor 1
CTLA-4: cytotoxic T-lymphocyte-associated protein 4
Ptpn2: tyrosine–protein phosphatase non-receptor type 2
dsRNA: double-stranded RNA
ADAR1: adenosine deaminase acting on RNA
MDA5: melanoma differentiation-associated protein 5
HIP1R: huntingtin interacting protein 1 related protein
VEGF: vascular endothelial growth factor
PTEN: phosphatase and tensin homolog
PI3K: phosphatidylinositol 3-kinase
DnMt1: DNA methyltransferase
OS: overall survival
LUAD: lung adenocarcinoma
PAK4: p21-activated kinase 4
EMT: epithelial–mesenchymal transition
TDO: tryptophan-2,3-dioxygenase
IDO: indoleamine 2,3-dioxygenase
GCN2: control non-depressible 2
MT: 1-methyltryptophan 1
ACAT1: acetyl-CoA acetyltransferase
MDSCs: myeloid derived suppressor cells
TAM: tumor-associated macrophages
CSF-1: colony-stimulating factor 1
FcγR: Fcγ receptor
TIM-3: T-cell immunoglobulin 3
TIGHT: T-cell immunoglobulin and ITIM domain
AP-1: activator protein 1
NF-κB: nuclear factor-κB
LXR: liver X receptor
AMP: adenosine monophosphate.
